# Effects of sertraline and sorafenib on HepG2 cells with a possible link to autophagy

**DOI:** 10.3892/mi.2025.246

**Published:** 2025-05-29

**Authors:** Gamze Demirel, Nil Pinarer, Mert Emre Ergin, Zeynep Saraçoğlu, Ceren Bingöl, Zeynep Güneş Özünal, Yaprak Dönmez Çakil

**Affiliations:** 1 Cancer and Stem Cell Center, Maltepe University, 34858 Istanbul, Türkiye; 2 Faculty of Medicine, Maltepe University, 34858 Istanbul, Türkiye; 3 Department of Medical Pharmacology, Faculty of Medicine, Atlas University, 34403 Istanbul, Türkiye; 4 Department of Medical Biology and Genetics, Faculty of Medicine, Maltepe University, 34858 Istanbul, Türkiye

**Keywords:** autophagy, liver cancer, sertraline, sorafenib, microscope

## Abstract

Liver cancer is one of the leading causes of cancer-related mortality worldwide. The current range of treatment options for patients with advanced-stage disease, including the first approved systemic therapy, sorafenib, has been demonstrated to have limited efficacy. A significant number of patients develop resistance to sorafenib treatment or discontinue its use due to adverse effects. Depression is a common complication of cancer, and the antidepressant, sertraline, has recently garnered considerable attention due to its anticancer activity. Accumulating evidence suggests that autophagy may represent a highly promising target for cancer therapy. Previously, the authors demonstrated that the sorafenib and sertraline combination exerted a synergistic anticancer effect on HepG2 cells. The present study examined the intracellular localization and mRNA expression levels of key autophagy markers, including Beclin-1, p62 and LC3, as well as the formation of acidic vesicular organelles using acridine orange staining, to further elucidate the link between autophagy and combined treatment of HepG2 cancer cells with sorafenib and sertraline. Drug treatment did not result in significant alterations in the expression levels of the *LC3* and *Beclin-1* genes. However, following treatment with sertraline, sorafenib, or both, the development of acidic vesicular organelles and the noticeable formation of LC3 and p62 puncta demonstrated the induction of changes related to autophagic activity. On the whole, the results of the present study support the effects of sertraline and sorafenib, which may, at least in part, be linked to autophagy.

## Introduction

Liver cancer was the seventh leading cause of cancer-related mortality in 2022, contributing to over a quarter million fatalities worldwide (1). Hepatocellular carcinoma (HCC) is its most common form, accounting for 75-85% of all cases. However, >50% of HCC cases are diagnosed at an advanced stage, where treatment options are limited (2).

As the first-line treatment for advanced-stage HCC, sorafenib is an oral multikinase inhibitor that suppresses tumor proliferation and induces apoptosis. Despite its initial efficacy, numerous patients develop resistance or experience severe side-effects, leading to the discontinuation of treatment (2-4). As a well-known treatment for psychiatric disorders, sertraline has exhibited promising results in cancer therapy due to its antitumor properties via apoptosis- and autophagy-related effects, and synergistic effects with other drugs (5-7). The antitumor properties of sertraline were first identified by Telerman *et al* in 1993(8).

As a mechanism characterized by the lysosomal degradation of intracellular proteins and organelles, autophagy has attracted significant attention regarding its role in human diseases and physiology. Autophagy can promote or inhibit cancer development, as well as the progression and response to therapy (9). During the early stages of tumorigenesis, autophagy functions as a protective mechanism for the body, limiting cancer development. However, in the advanced stages, autophagy enables malignant cells to survive under stress conditions, such as the hypoxic tumor microenvironment and therapy-induced starvation (10).

Autophagy is regulated by proteins, such as Beclin-1, p62/sequestosome1 (SQSTM1) and autophagy-related protein (ATG)8/LC3, which are involved in the formation and maturation of autophagosomes. These autophagosomes fuse with lysosomes for the degradation of cellular debris (11). Recent observations suggest that sorafenib induces autophagy in liver cancer cells by modulating several signaling pathways, including the mammalian target of rapamycin (mTOR) and SHP-1/STAT3/MCL-1/Beclin-1 pathways, as well as by modulating endoplasmic reticulum stress induction, sphingolipid metabolism imbalance and microRNA transcription alteration (12). The dysregulation of autophagy has also been linked to sertraline in several cell lines (4-7). Sertraline has been shown to induce autophagy via the activation of AMP-activated protein kinase (AMPK), which inhibits the mechanistic target of the mTOR-ribosomal protein S6 kinase B1 signaling pathway (13). On the other hand, contradictory results have been reported in lung cancer, where sertraline inhibits autophagy and facilitates TRAIL-induced apoptosis (14).

The authors have previously reported that sorafenib and sertraline exert a synergistic anticancer effect on HepG2 cells, significantly reducing cell viability and inducing apoptosis at lower doses compared to each drug used alone (15). To further investigate the effects of sorafenib and sertraline, and investigate a possible link between autophagy and combined treatment with both agents in HepG2 cells, the present study examined the mRNA expression levels and cellular localizations of the key autophagy markers and the formation of acidic vesicular organelles.

## Materials and methods

### Cells, cell culture and treatments

HepG2 cells (cat. no. HB-8065, American Type Culture Collection). were maintained in Dulbecco's modified Eagle's medium (DMEM, MilliporeSigma) containing 1% antibiotics (10 mg/ml streptomycin and 10,000 U/ml penicillin, PAN-Biotech GmbH) and 10% fetal bovine serum (FBS, Biosera) in an incubator with 5% CO_2_ at 37˚C. The monitoring of cultured HepG2 cells was performed using the Zeiss PrimoVert inverted phase contrast microscope (Zeiss AG). For starvation, cells were incubated in Dulbecco's phosphate-buffered saline (DPBS, Gibco; Thermo Fisher Scientific, Inc.) for 2 h in an incubator at 37˚C (16).

Sorafenib (LC Laboratories) and sertraline (MilliporeSigma) were dissolved in dimethyl sulfoxide (DMSO) and distilled water, respectively, to a concentration of 10 mmol/l. The drugs were treated as previously reported with IC_50_/2 doses for 24 h (17.8 µl sorafenib and 8.9 µl sertraline) (15).

### Total RNA extraction

Following a 24-h incubation period at 37˚C with the drugs in question, whether alone or in combination, the cells were trypsinized and washed with PBS. RNA extraction was then performed using a Thermo Scientific GeneJET RNA Purification kit (cat. no. K0731, Thermo Fisher Scientific, Inc.), as per the manufacturer's instructions. The concentration and purity of the extracted RNA samples were subsequently assessed with a BioTek Synergy Microplate Reader (Agilent Technologies, Inc.), utilizing UV absorbance.

### Complementary DNA (cDNA) synthesis and reverse transcription-quantitative PCR (RT-qPCR)

The Thermo Scientific RevertAid First Strand cDNA Synthesis kit (cat. no. K1621, Thermo Fisher Scientific, Inc.) was employed to synthesize cDNA from total RNA, following the instructions provided by the manufacturer. qPCR was conducted on a LightCycler^®^ 96 instrument (Roche Diagnostics) using the Ampliqon RealQ Plus Master Mix Green Without ROX kit (cat. no. A323402, Ampliqon A/S) and gene-specific primers for *Beclin-1* (NM_003766; forward sequence, 5'-CTGGACACTCAGCTCAACGTCA-3' and reverse sequence, 5'-CTCTAGTGCCAGCTCCTTTAGC-3'); *LC3* (NM_022818; forward sequence, 5'-GAGAAGCAGCTTCCTGTTCTGG-3' and reverse sequence, 5'-GTGTCCGTTCACCAACAGGAAG-3') and GAPDH (forward sequence, 5'-ATGGGTGTGAACCATGAGAA-3' and reverse sequence, 5'-GTGCTAAGCAGTTGGTGGTG-3'). qPCR was performed using with the following thermocycling conditions: initial denaturation at 95˚C for 10 min, followed by 40 cycles of denaturation at 95˚C for 15 sec, annealing at 60˚C for 30 sec, and extension at 72˚C for 30 sec. qPCR primer sequences were obtained from OriGene Technologies, Inc. The 2^-^^ΔΔ^^Cq^ was employed for the relative quantification of gene expression, with *GAPDH* serving as the internal control (17). The untreated control was used as a calibrator to determine whether DMSO influences the expression of related genes.

### Confocal laser scanning microscopy

Autoclaved coverslips were introduced into the wells of a 12-well plate, with 100,000 cells seeded per well. On the subsequent day, the cells were treated with sertraline and sorafenib, either individually or in combination, and incubated for 24 h. The medium of the starvation control group was washed twice, replaced with DPBS, and incubated at 37˚C for 2 h. A stock solution of acridine orange (Thermo Fisher Scientific, Inc.) was prepared in water at a concentration of 1 mg/ml and stored at 4˚C. The staining was conducted with acridine orange (cat. no. A6014, MilliporeSigma). at a final concentration of 1 µg/ml for 20 min at 37˚C following fixation of the cells with 4% paraformaldehyde (cat. no. P6148, MilliporeSigma) for 15 min at room temperature. The cells were then washed three times with PBS and imaged using a Zeiss LSM 700 confocal microscope (Zeiss AG). The excitation laser for green fluorescence was 473 nm, and for red fluorescence, it was 559 nm. The emission filters were 520 and 572 nm, respectively.

Following the treatments and subsequent fixation of the cells, they were also labeled with p62 (1:200; cat. no. A19700, ABclonal Biotech Co., Ltd.) and LC3 (1:50; cat. no. A5618, ABclonal Biotech Co., Ltd.) antibodies. Incubation was performed at 4˚C overnight. The cells were incubated with a 0.1% Triton X-100/PBS solution for permeabilization for 10 min. The cells were then incubated at 37˚C with PBS containing 1% BSA for 30 min to block non-specific binding. Specific primary antibodies were prepared at a dilution of 1:50 for p62 and 1:200 for LC3, and the cells were incubated overnight at 4˚C. The following day, the cells were washed three times with PBS and then incubated for 1 h at room temperature in the dark with Alexa Fluor 488-conjugated (1:200; cat. no. A-11008, Invitrogen; Thermo Fisher Scientific, Inc.) and Alexa Fluor 555-conjugated (1:200; cat. no. A-21428, Invitrogen; Thermo Fisher Scientific, Inc.) anti-rabbit secondary antibodies. After staining, the cells were washed three times with PBS and incubated with DAPI (cat. no. D1306, Thermo Fisher Scientific, Inc.) solution (1 µg/ml) for 10 min for nuclear staining. The preparations were mounted with a mounting medium and imaged using a Zeiss LSM700 confocal microscope (Zeiss AG).

### Statistical analysis

The bar graphs were generated, and the statistical analysis was performed using GraphPad Prism 9 Software (Dotmatics). A one-way analysis of variance (ANOVA) was conducted to determine the difference between the means of the groups, followed by Tukey's multiple comparisons test to assess pairwise differences. The data are presented as the mean ± standard error of mean (SEM). A P-value <0.05 was considered to indicate a statistically significant difference.

## Results

### Effects of sertraline and sorafenib on LC3 and Beclin-1 gene expression levels

**Figure 1:**
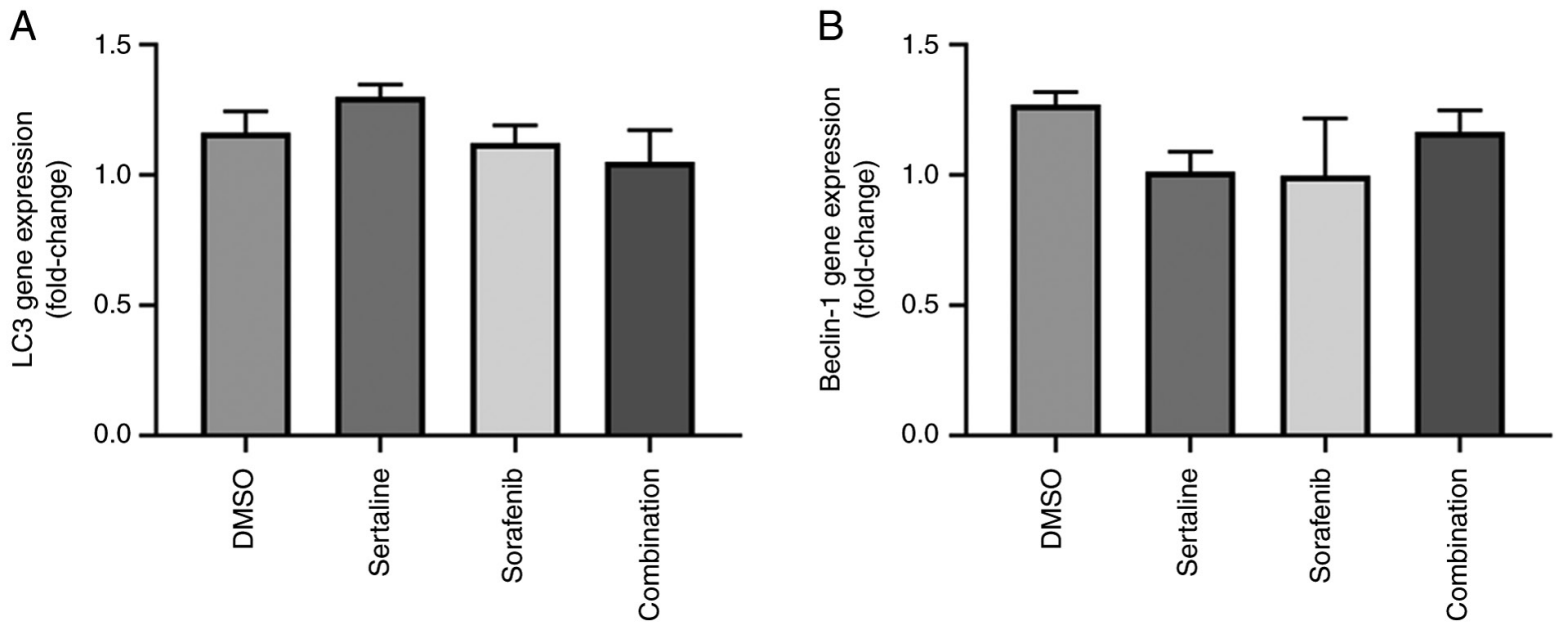
Bar graphs demonstrating (A) *LC3* gene expression, and (B) *Beclin-1* gene expression in HepG2 cells treated with DMSO as a vehicle control, sertraline and sorafenib, and the combination of sertraline and sorafenib for 24 h. Error bars represent the mean ± SEM. Statistical analysis using one-way ANOVA revealed no significant differences among the groups (P=0.0916 for *LC3* and P=0.6022 for *Beclin-1*).

The effects of sertraline and sorafenib on *LC3* and *Beclin-1* gene expression levels are demonstrated in Fig. 1. No significant differences were found between the groups for both genes. The drugs did not induce a significant change in the *LC3* and *Beclin-1* gene expression levels (P=0.0916 for LC3 and P=0.6022 for Beclin-1).

### Effects of sertraline and sorafenib on the formation of acidic vesicular organelles

**Figure 2:**
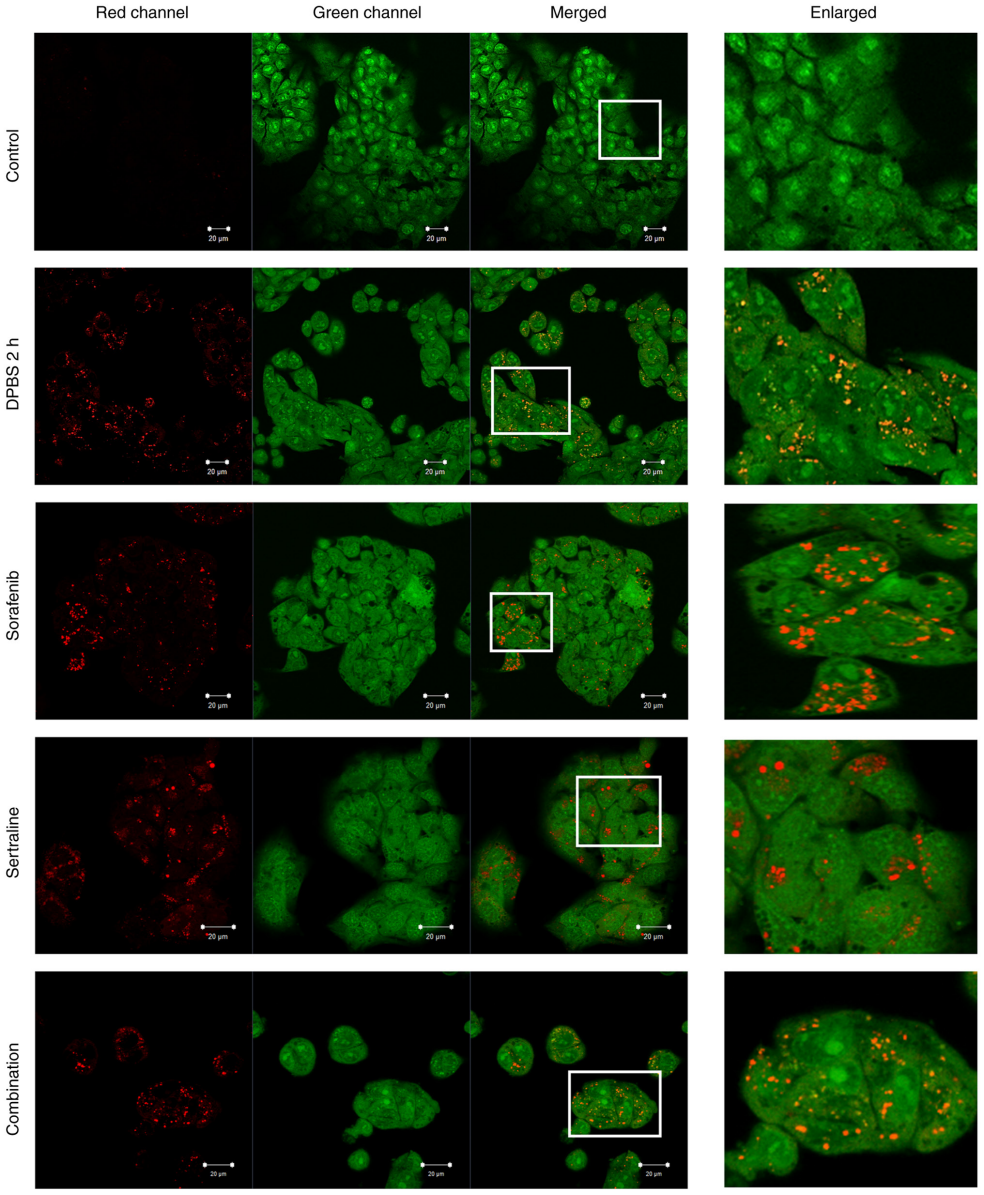
Acridine orange staining detected in red and green fluorescent channels for the HepG2 cells in the control, starvation control (incubated in DPBS for 2 h to induce starvation), sertraline, sorafenib, and the sertraline and sorafenib combined treatment groups. Scale bars, 20 µm. Magnification, x40. The images in the ‘Enlarged’ panel are a magnification of the boxed area in the ‘Merged’ image panel (enlarged image scale bar, 5 µm).

The formation of acidic vesicular organelles following treatment with sertraline, sorafenib, or a combination of both was evaluated using acridine orange staining and compared to a starvation control group that was incubated in DPBS for 2 h, as demonstrated in Fig. 2. In the starvation control, acridine orange formed aggregates that emitted bright red fluorescence, indicating the acidic compartments. Aggregate formation was also evident following sertraline, sorafenib and combination treatments. Conversely, such aggregates were not observed in the control cells (Fig. 2).

### Effects of sertraline and sorafenib on the localization of LC3 and p62

**Figure 3:**
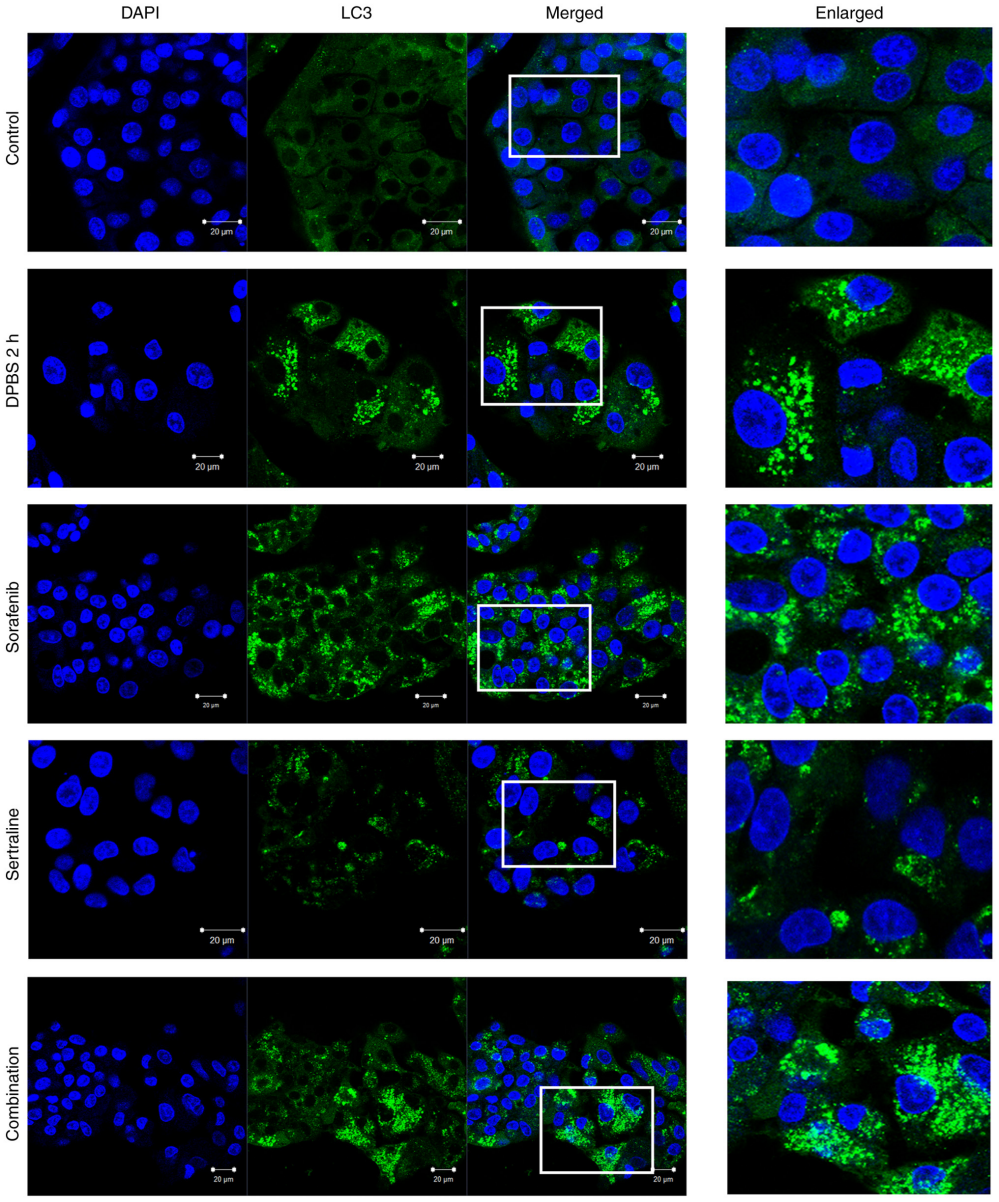
LC3 staining detected in the HepG2 cells in the control, starvation control (incubated in DPBS for 2 h to induce starvation), sertraline, sorafenib, and the sertraline and sorafenib combined treatment groups. Fixed and permeabilized cells were stained with anti-LC3 and Alexa Fluor 488-conjugated anti-rabbit secondary antibodies. Nuclei were counterstained using DAPI stain. Scale bars, 20 µm. Magnification, x40. The images in the ‘Enlarged’ panel are a magnification of the boxed area in the ‘Merged’ image panel (enlarged image scale bar, 5 µm).

**Figure 4:**
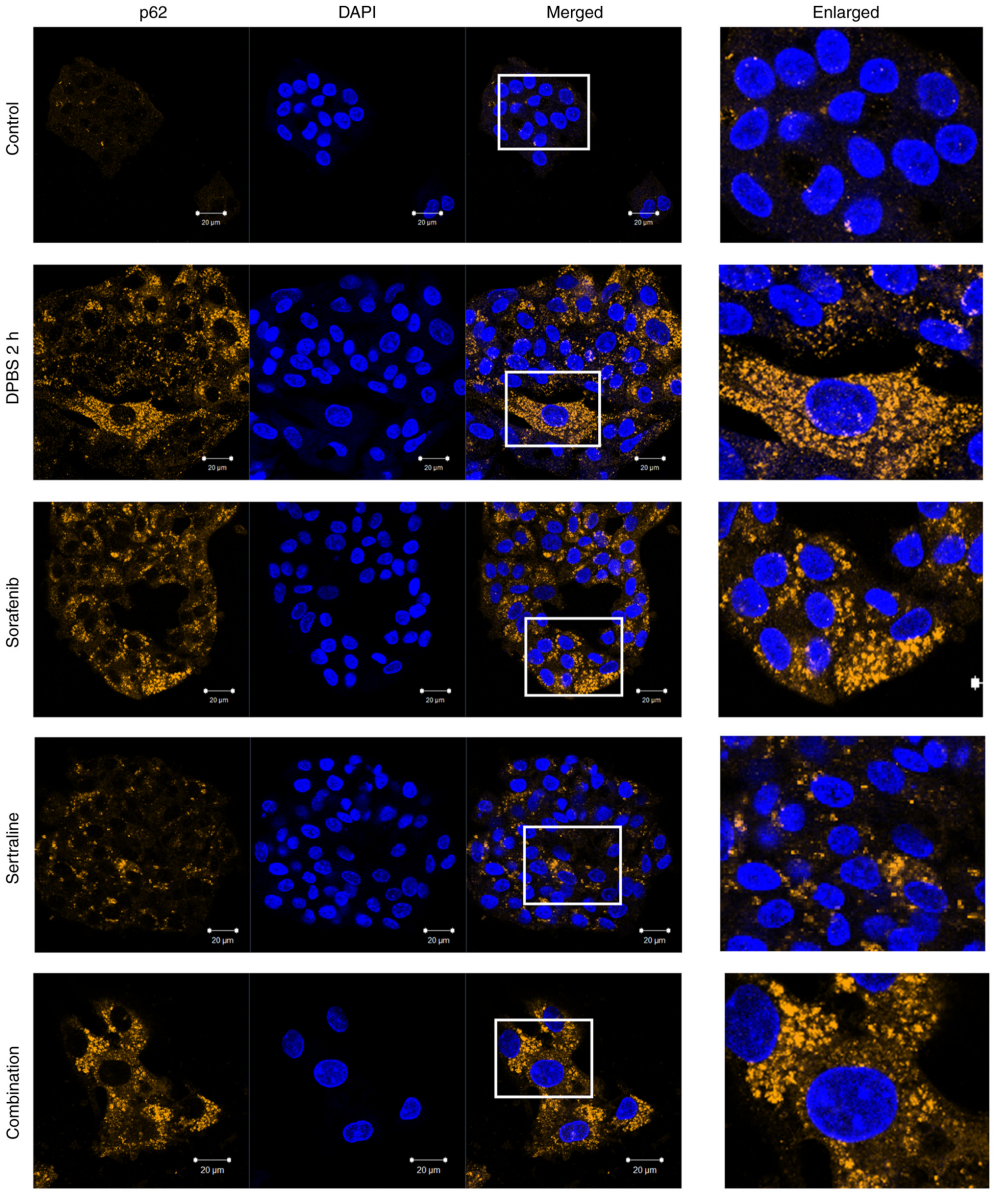
p62 staining detected in the HepG2 cells in the control, starvation control (incubated in DPBS for 2 h to induce starvation), sertraline, sorafenib, and the sertraline and sorafenib combined treatment groups. Fixed and permeabilized cells were stained with anti-p62 and Alexa Fluor 550-conjugated anti-rabbit secondary antibodies. Nuclei were counterstained using DAPI stain. Scale bars, 20 µm. Magnification, x40. The images in the ‘Enlarged’ panel are a magnification of the boxed area in the ‘Merged’ image panel (enlarged image scale bar, 5 µm).

The autophagic activity was further evaluated by detecting LC3 and p62 localization using immunofluorescence staining. As illustrated in Fig. 3, clear LC3 puncta were observed, indicating autophagosome formation in the starvation control cells and drug-treated cells. A plurality of spots was particularly observed in cells treated with sorafenib or sertraline-sorafenib combination therapy. A similar pattern was obtained for the appearance of p62 puncta in starvation-induced cells and drug-treated cells (Fig. 4). By contrast, LC3 and p62 puncta formations were not evident in the untreated control group.

## Discussion

A significant number of patients are diagnosed with advanced liver cancer and, regrettably, do not derive long-term benefit from systemic therapy due to the adverse effects and development of drug resistance through a number of different mechanisms (18). The mechanisms contributing to a decreased response to sorafenib include the phosphoinositide 3-kinase (PI3K)/protein kinase B (Akt) and Janus kinase-signal transducer and activator of transcription (JAK-STAT) pathways, the inhibition of pro-apoptotic signals, the presence of cancer stem cells, epithelial-mesenchymal transition, and hypoxia-driven responses (19). The administration of combination treatments within the context of drug repurposing may facilitate the attainment of superior outcomes for cancer patients. The antidepressant, sertraline, was previously demonstrated to exhibit promising anticancer effects, both when administered alone and in combination with other agents, across a range of cancer cell lines (20). Previously, the authors also demonstrated that sorafenib and sertraline exerted a synergistic anticancer effect in HepG2 cells, resulting in a significant reduction in cell viability and increased apoptosis at lower concentrations compared to treatment with each drug separately (15). The present study aimed to further investigate the effects of sorafenib and sertraline with a possible link to autophagy in HepG2 cells.

The regulation of autophagy is considered a viable strategy in cancer therapy. Autophagy is an essential process for maintaining cellular homeostasis, and it involves the degradation and recycling of cellular components (21). Various conditions can trigger autophagy, including nutrient deprivation, growth factor withdrawal, hypoxia, or drug treatment (22). Accordingly, in the present study, autophagy was induced by nutrient deprivation by incubating the cells in DPBS for 2 h,, which served as a positive control alongside drug treatment.

The autophagy process begins with the formation of the isolation membrane, known as the phagophore, which elongates to engulf cytoplasmic components. The assembly and formation of autophagosomes rely on the coordinated action of multiple functional units, including the ULK1 complex, the PI3K complex, the ATG9A system, and the ATG12- and LC3-conjugation systems (23). Initially, LC3 exists in the precursor form, which is cleaved by the enzyme ATG4 to produce its cytosolic form LC3-I. Subsequently, ATG7 and ATG3 attach phosphatidylethanolamine (PE) to LC3-I, converting it into LC3-II, which is then directed to the developing autophagosome (24). This conversion is critical for autophagy advancement and membrane structure stability, rendering LC3 a prominent autophagic marker. When the autophagosome is fully formed, PE is removed by ATG4, and LC3 is then released back into the cytosol (25).

p62/SQSTM1 is a cargo receptor that plays a pivotal role in the intersection between the ubiquitin-proteasome system and autophagy by recognizing ubiquitinated proteins destined for autophagic destruction (26). As p62 accumulates in autophagosomes, it can serve as an indicator of autophagic activity. A direct interaction between p62 and LC3 facilitates the degradation of ubiquitinated protein aggregates by autophagy (27). The present study demonstrated clear LC3 puncta in the cytoplasm of sertraline, sorafenib and combination treatmetn groups, indicating an increase in the number of autophagosomes following the respective treatments, similar to that in the starvation group compared to the control group.

However, while a common approach to assessing autophagy involves counting LC3 puncta or autophagosomes, an increase in the number of autophagosomes does not necessarily indicate enhanced autophagy, as it could also suggest a blockage in the process. The majority of assays utilize LC3 as a model substrate to measure autophagic flux. It is important to determine the extent to which LC3-II is degraded in a lysosome-dependent manner over a specified period (28). Moreover, given that autophagy is a multistep process, the measurement of LC3 or p62 alone is insufficient to provide a comprehensive understanding of the cellular events that occur (29). This is regarded as a limitation of the present study.

Acridine orange is a cell-permeable green fluorophore that accumulates in acidic vesicular organelles by protonation. Depending on the concentration, it exhibits a metachromatic shift to red fluorescence (30). Consequently, red fluorescence can be observed in acidic vesicular organelles such as autolysosomes. For the purpose of leveraging this property, acridine orange was used to measure the increase in acidic vesicular organelle volume during autophagy induction. The findings of the present study regarding the use of acridine orange were in accordance with the results for LC3 and p62. This demonstrated that changes related to autophagic activity were induced in the sertraline, sorafenib and combination treatment groups.

Although sorafenib was demonstrated to induce autophagy in liver cancer, it was also shown that autophagy triggered by liver cancer cells that have developed resistance to sorafenib could contribute to the emergence of further drug resistance. The effect of sertraline on autophagy has been found to vary depending on the cell type (31). While some studies have shown that it induces the autophagic flux (6,7,13,32), others have reported that it inhibits autophagy (13). To the best of our knowledge, the present study is the first to demonstrate the LC3 and p62 puncta, indicating an increase in the number of autophagosomes in sertraline-treated HepG2 cells. Furthermore, when applied in combination with sorafenib, it appears to introduce changes related to the autophagic flux effectively. These findings suggest that sertraline may play a dual role in cancer therapy, acting as a supportive agent due to its antidepressant effects, while having the ability to modulate autophagy in addition to its other anticancer activities (20). Jiang *et al* (6) reported that sertraline induced autophagy and inhibited cell growth, leading exclusively to autophagic cell death without triggering caspase-mediated apoptosis. In non-small cell lung cancer cells, the combination of sertraline and erlotinib enhanced autophagy activation and tumor cell death through the mutual regulation of the AMPK/mTOR/S6K pathways. However, when autophagy was inhibited, sertraline alone or in combination with erlotinib was less effective (6). Hwang *et al* (13) reported the role of sertraline in AMPK-MTOR signaling-mediated autophagy (12). Moreover, recent research suggests that sertraline targets prostate cancer stem cells by regulating redox balance and activating apoptotic and autophagic signaling pathways (32). Contradictory results were observed in TRAIL-resistant lung cancer cells. Zinnah *et al* (14) reported that, by inhibiting autophagy, sertraline reduced AMPK phosphorylation and increased death receptor 5 expression, facilitating TRAIL-induced apoptosis (14).

In conclusion, the present study demonstrates that the combination of sorafenib and sertraline induces changes related to autophagic activity in HepG2 cells. While the autophagy-inducing effects of sorafenib are well-documented, variable effects of sertraline on autophagy in different types of cancer have been demonstrated. The present study revealed that sorafenib and the antidepressant, sertraline, when applied alone or in combination, resulted in an increase in the number of autophagosomes compared to the control group, thereby introducing changes related to autophagic activity in HepG2 cells. Future studies are required to explore the molecular mechanisms underlying the effects of sorafenib and sertraline in order to obtain a more in-depth understanding of the clinical applicability of this combination in addressing treatment resistance and improving patient outcomes.

## Data Availability

The data generated in the present study may be requested from the corresponding author.
